# Anti-Obesity and Hypoglycemic Effects of *Poncirus trifoliata* L. Extracts in High-Fat Diet C57BL/6 Mice

**DOI:** 10.3390/molecules21040453

**Published:** 2016-04-06

**Authors:** Sheng Jia, Zhiwei Gao, Shuxia Yan, Yanhong Chen, Chongde Sun, Xian Li, Kunsong Chen

**Affiliations:** 1 Zhejiang Provincial Key Laboratory of Horticultural Plant Integrative Biology, Zhejiang University, Zijingang Campus, Hangzhou 310058, China; shengjia@zju.edu.cn (S.J.); arillaysx@zju.edu.cn (S.Y.); adesun2006@zju.edu.cn (C.S.); akun@zju.edu.cn (K.C.); 2 Laboratory of Fruit Quality Biology, Zhejiang University, Zijingang Campus, Hangzhou 310058, China; 3 Vascular Surgery, Second Affiliated Hospital of Nanjing Medical University, Changzhou 213003, China; dr_gaozw@126.com; 4 Laboratory Animal Center of Zhejiang University, Hangzhou 310058, China; chenyanhong@zju.edu.cn

**Keywords:** anti-obesity, hypoglycemic, *Poncirus trifoliata*, obesity, diabetes

## Abstract

The present study investigated the possible anti-obesity and hypoglycemic effects of *Poncirus trifoliata* L. extracts. Mature fruit were divided into flavedo (PF) and juice sacs (PJ), and extracts from them were tested on C57BL/6 mice fed a high-fat diet (HFD) for thirteen weeks. Both fruit extracts (40 mg/kg body weight, respectively) showed anti-obesity and hypoglycemic effects. Consumption of PF and PJ extracts reduced body weight by 9.21% and 20.27%, respectively. Liver and adipose weights, fasting glucose, serum triglyceride (TG), and low density lipoprotein cholesterol (LDL-c) levels decreased significantly, while serum high density lipoprotein cholesterol (HDL-c) and oral glucose tolerance levels increased significantly in response to two fruit extracts. These effects were due in part to the modulation of serum insulin, leptin, and adiponectin. Furthermore, transcript levels of fatty acid synthase (*FAS*) and stearoyl-CoA desaturase 1 (*SCD1*) were reduced while those of carnitine palmitoyltransferase 1α (*CPT1α*) and insulin receptor substrate 2 (*IRS2*) were increased in the liver of C57BL/6 mice, which might be an important mechanism affecting lipid and glucose metabolism. Taken together, *P. trifoliata* fruit can be potentially used to prevent or treat obesity and associated metabolic disorders.

## 1. Introduction

Noninfectious chronic diseases (NCDs) such as obesity and diabetes have emerged as a global health problem. In 2014, according to World Health Organization, more than 1.9 billion adults were estimated to be overweight, with over 600 million being obese [1]. Development of obesity is usually accompanied with other metabolism disorders such as hyperlipidemia, diabetes and inflammation [2]. Many NCDs interact with each other and developments of different NCDs are usually multifactorially affected. Dietary intervention is an important strategy to prevent and control these metabolic syndrome related NCDs since there are many structurally diverse food-derived molecules targeting different metabolism pathways *in vivo* [3,4].

Edible plants provide diverse bioactive compounds, which can be used for efficient prevention and treatment of the multifactorial NCDs. For example, anthocyanin from mulberry and cherry prevented oxidative stress and inflammation in diet-induced obese C57BL/6 mice [5]. Neohesperidin from *Citrus aurantium* had hypolipidemic and hypoglycemic effects in KK-A^y^ mice [6]. *Poncirus trifoliata* L., a member of the Rutaceae family, has been cultivated in China for more than 2000 years, and is a commonly used rootstock for the *Citrus* genus. The fruit has diverse nutritional properties, and it has a long history of use as a Chinese medicine—for example, as a potential anti-leukemic candidate since it induced apoptosis in human promyelocytic leukemia HL-60 cells [7] and for the anti-inflammatory activity of compounds in fruit extracts [8]. Lee *et al.* reported that neohesperidin and poncirin isolated from the fruit have protective effects on potential gastric disease [9] and aqueous extract of immature fruit of *P. trifoliata* suppressed body weight gain in Sprague–Dawley rats [10]. The bioactivities in fruit extracts are due to the various compounds, which accumulate differently in various fruit tissues and developmental stages, but to date there has been very few studies on anti-obesity and hypoglycemic effects of different tissues of mature *P. trifoliata* fruit.

In the present study, the possible anti-obesity and hypoglycemic effects of *P. trifoliata* flavedo (PF) and juice sac (PJ) extracts were investigated. Anti-obesity and metabolic-related activities were evaluated by using the high-fat diet (HFD) C57BL/6 mice model.

## 2. Results

### 2.1. LC-MS Identification and HPLC Quantification

Four flavanones were identified using LC-MS, *i.e.*, narirutin and its isomer naringin, and didymin and its isomer poncirin (Table 1). Naringin and poncirin had neohesperidose in R1, while the other two had rutinose in R1 (Table 1).

**Figure 1:**
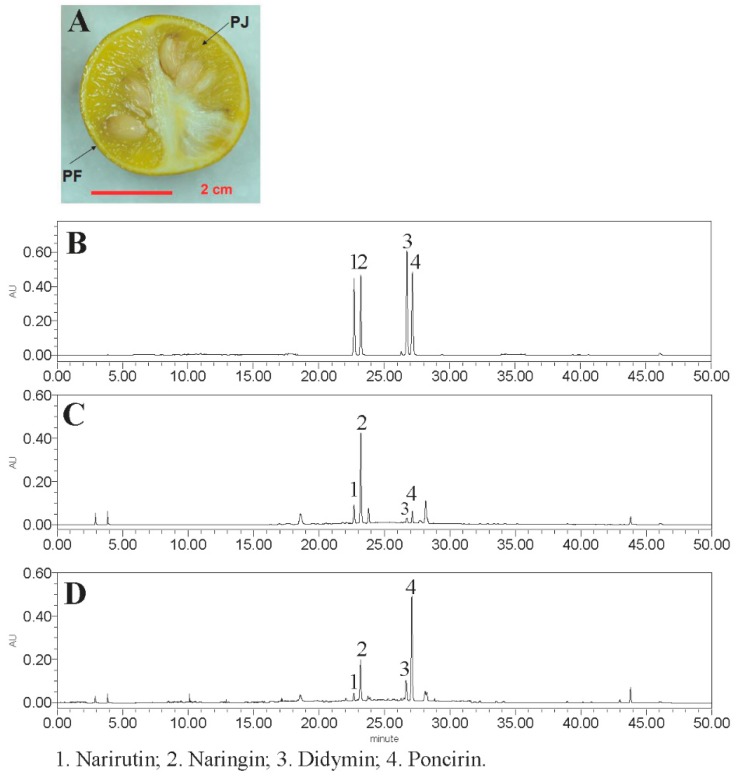
*P. trifoliata* fruit (**A**) and HPLC chromatograms of four flavanone standards (**B**); *P. trifoliata* flavedo (PF) (**C**); and juice sacs (PJ) (**D**) extracts (λ = 280 nm).

Quantification of four flavanones in *P. trifoliata* extracts was accomplished by HPLC (Figure 1), which was carried out according to the retention time and peak area compared with their standards. PJ extract had higher amounts of the four flavanones (184.91 mg/g) compared to PF (99.21 mg/g) and the major flavanones were naringin in PF (77.05 ± 8.16 mg/g) and poncirin in PJ (107.46 ± 12.82 mg/g) (Table 2).

### 2.2. Effects of P. trifoliata Extracts on Lipid Related Parameters

**Figure 2:**
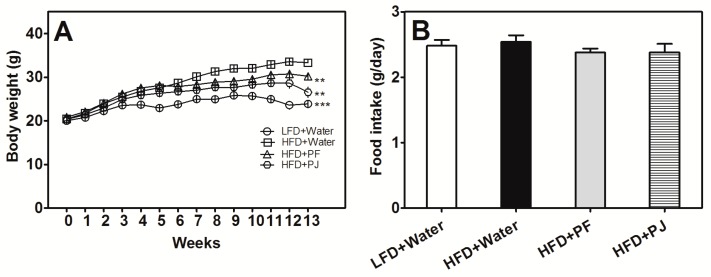
Effects of PF and PJ extracts (40 mg/kg body weight, respectively) on body weight (**A**) and food intake (**B**) in high-fat diet (HFD) C57BL/6 mice (13 weeks). Data are presented as mean ± SEM (*n* = 6–10). ** *p* < 0.01, *** *p* < 0.001, compared to the HFD group treated with water.

At the end of the animal experiment (13-weeks), the body weight of HFD mice was 33.30 ± 0.84 g, which was significantly higher than that of the low-fat diet (LFD) mice (23.89 ± 0.69 g) at the same stage (Figure 2A). Treatments of the HFD mice with both PF and PJ resulted in significantly decreased body weight, reduced by 9.21% (30.23 ± 0.34 g) and 20.27% (26.55 ± 1.20 g), respectively, at week 13 (Figure 2A). No significant difference was observed in food intake of the different groups (Figure 2B). In addition, the weights of liver, epididymal adipose, and perirenal adipose were significantly reduced in the groups treated with fruit extracts, compared to the HFD control group (*p* *<* 0.05, *p* *<* 0.01, *p* *<* 0.001) (Table 3).

Serum, triglyceride (TG) increased significantly in HFD control mice (1.95 ± 0.35 mmol/L) compared to the LFD group (1.00 ± 0.09 mmol/L) and PF and PJ treatments resulted in significantly lower TG, *i.e.*, 0.888 ± 0.05 mmol/L and 0.64 ± 0.03 mmol/L, respectively (Table 3). In addition, HFD control mice showed significantly lower high-density lipoprotein cholesterol (HDL-c) (1.42 ± 0.20 mmol/L) and higher low-density lipoprotein cholesterol (LDL-c) (0.65 ± 0.06 mmol/L), when compared with the LFD group. Both fruit-extract-treated HFD groups showed significantly lower LDL-c (*p* *<* 0.05, *p* *<* 0.01) and improved levels of HDL-c (*p* *<* 0.01), although there was no significant difference in serum total cholesterol (TCH) between HFD and LFD groups or within the HFD groups (Table 3).

**Figure 3:**
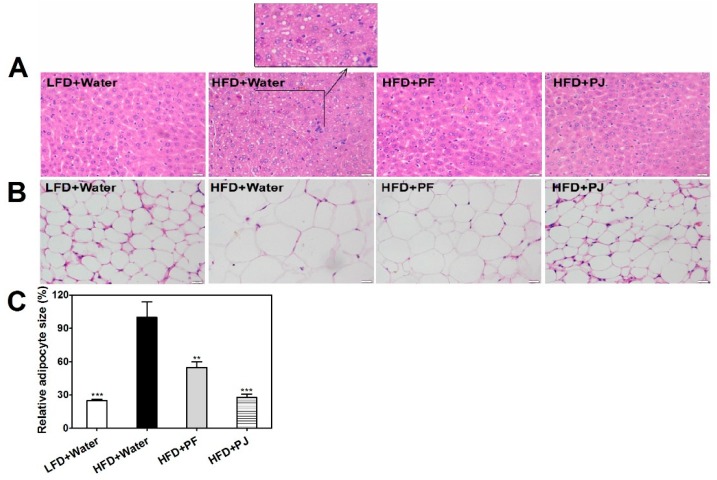
Effects of PF and PJ extracts on morphological changes (×200) in liver (**A**) and epididymal adipose (**B**); and relative adipocyte size (**C**) in HFD C57BL/6 mice. Data are presented as mean ± SEM (*n* = 6–10). ** *p* < 0.01, *** *p* < 0.001, compared to the HFD group treated with water.

Histological observation showed intense lipid accumulation in the liver of HFD mice treated with water, compared with the LFD mice (Figure 3A). Both PF and PJ treatments markedly inhibited lipid accumulation in the liver of HFD mice (Figure 3A). Hypertrophy of the adipocytes was observed in the epididymal adipose tissue of the HFD mice treated with water, when compared with LFD mice (Figure 3B). This was attenuated by PF and PJ treatments, which resulted in decreased size of epididymal adipocytes in mice (*p* *<* 0.01, *p* *<* 0.001) (Figure 3B,C).

### 2.3. Effects of P. trifoliata Extracts on Glucose Related Parameters

**Figure 4:**
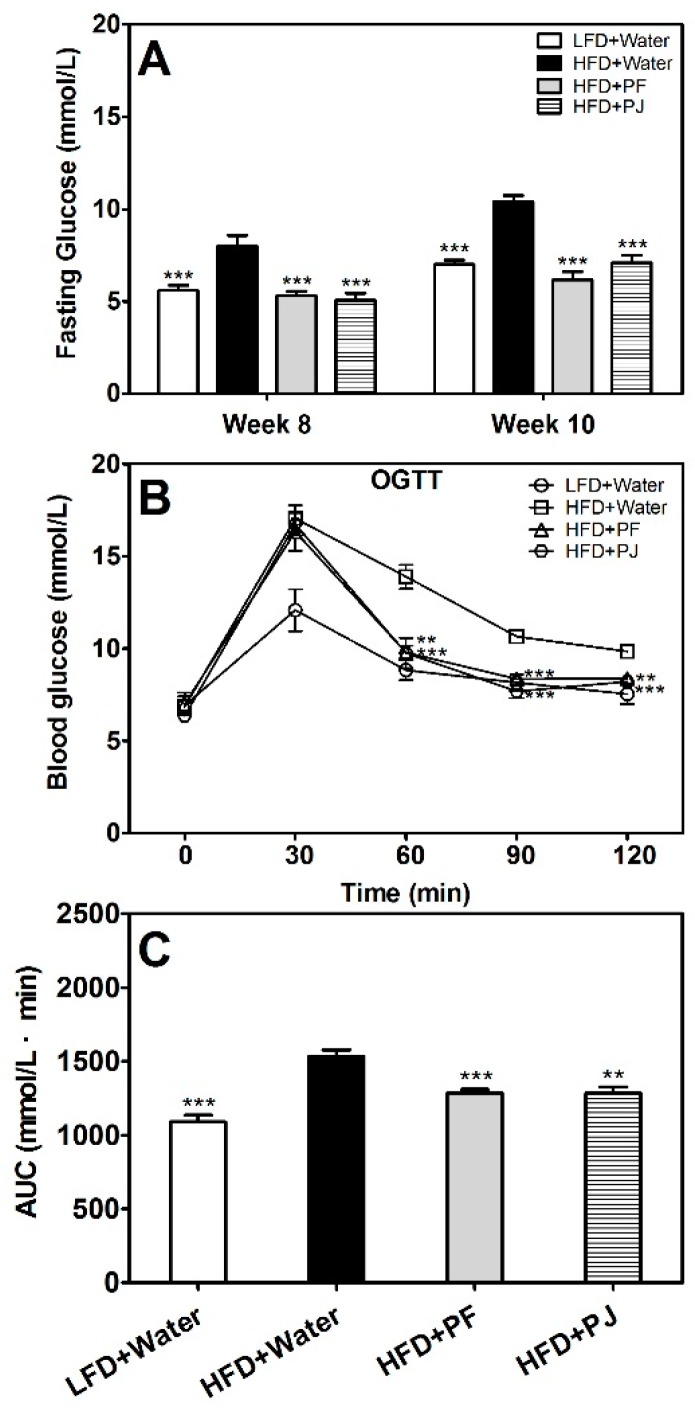
Effects of PF and PJ extracts on fasting glucose (**A**); oral glucose tolerance test (OGTT) (**B**); and area under curve (AUC) (**C**) in HFD C57BL/6 mice. Data are presented as mean ± SEM (*n* = 6–10). ** *p* < 0.01, *** *p* < 0.001, compared to the HFD group treated with water.

HFD mice treated with water began to show hyperglycemia symptoms from week 8, where the fasting glucose was 8.03 ± 0.54 mmol/L compared with that of the LFD mice (5.59 ± 0.28 mmol/L, *p* < 0.001) (Figure 4A). In week 10, fasting glucose of the HFD control group reached 10.40 ± 0.34 mmol/L, and 7.02 ± 0.21 mmol/L in LFD mice. PF and PJ treatments significantly decreased fasting glucose both in week 8 and week 10 (*p* < 0.001) (Figure 4A). After the oral glucose tolerance test (OGTT), the blood glucose levels remained high at 17.04 ± 0.73 mmol/L at 1 h and 9.84 ± 0.14 mmol/L at 2 h after the oral intake of glucose in HFD control group (Figure 4B), which indicated an apparent impairment of the glucose tolerance. Administration of either of the fruit extracts exerted significant effects on the glucose tolerance, where the blood glucose levels were reduced to 8.37 ± 0.29 mmol/L and 8.20 ± 0.23 mmol/L at 2 h, respectively, for the PF-treated and PJ-treated mice (Figure 4B,C).

### 2.4. Effects of P. trifoliata Extracts on Insulin, Leptin and Adiponectin

**Figure 5:**
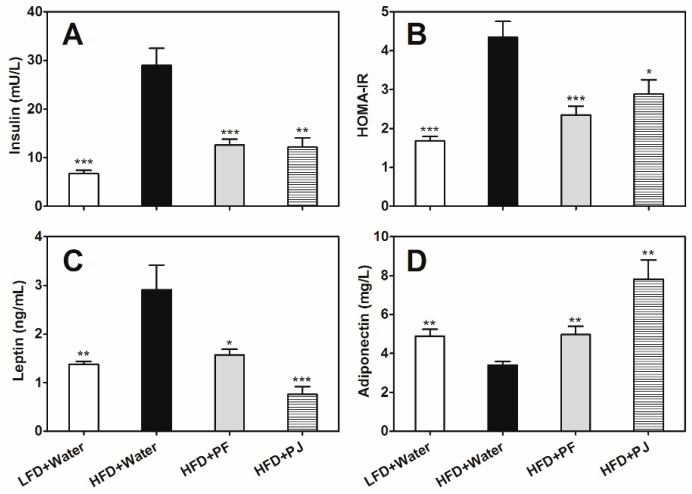
Effects of PF and PJ extracts on serum insulin (**A**); homeostasis model assessment of insulin resistance (HOMA-IR) (**B**); leptin (**C**); and adiponectin (**D**) in HFD C57BL/6 mice. Data are presented as mean ± SEM (*n* = 6–10). * *p* < 0.05, ** *p* < 0.01, *** *p* < 0.001, compared to the HFD group treated with water.

Compared to the LFD mice, the HFD mice showed hyperinsulinemic and insulin-resistant symptoms (Figure 5A,B). The insulin level in HFD control group was 28.97 ± 3.50 mU/L and the homeostasis model assessment of insulin resistance (HOMA-IR) level was 4.34 ± 0.41. PF and PJ treatments significantly decreased serum insulin (*p* < 0.01, *p* < 0.001) and HOMA-IR levels (*p* < 0.05, *p* < 0.001) in HFD mice (Figure 5A,B), indicating that they could ameliorate insulin resistance. Compared to the LFD mice, the HFD mice also showed high serum leptin (2.91 ± 0.51 ng/mL) and low serum adiponectin (3.39 ± 0.20 mg/L) levels (Figure 5C,D), but both PF and PJ treatments significantly decreased serum leptin levels (*p* < 0.05, *p* < 0.001) and increased serum adiponectin levels in the HFD mice (*p* < 0.01) (Figure 5C,D).

### 2.5. Gene Expression Analysis

**Figure 6:**
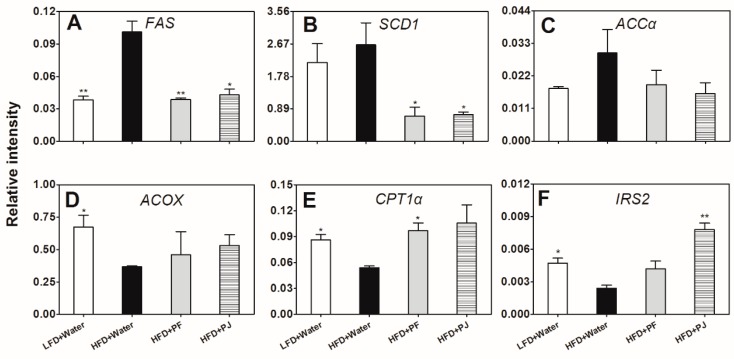
Effect of PF and PJ extracts on relative gene expression of *FAS* (**A**); *SCD1* (**B**); *ACCα* (**C**); *ACOX* (**D**); *CPT1α* (**E**); and *IRS2* (**F**) in livers of HFD C57BL/6 mice. Data are presented as mean ± SEM (*n* = 4). * *p* < 0.05, ** *p* < 0.01, compared to the HFD group treated with water.

The genes studied were fatty acid synthase (*FAS*), stearoyl-CoA desaturase 1 (*SCD1*), acetyl-CoA carboxylase α (*ACCα*), acyl-CoA oxidase (*ACOX*), carnitine palmitoyltransferase 1α (*CPT1α*), and insulin receptor substrate 2 (*IRS2*). For the genes involved in fatty acids biosynthesis, the transcript levels of *FAS* and *SCD1* in HFD mice were higher than in the LFD mice, while they were significantly reduced by both PF and PJ treatments in the HFD mice (Figure 6A,B). Meanwhile, the expression of *ACCα* showed no significant difference between HFD and LFD or among HFD groups (Figure 6C). For the genes involved in fatty acids oxidation, the expressions of both *ACOX* and *CPT1α* were significantly inhibited by HFD, when compared with the LFD (Figure 6D,E). PF treatment of HFD mice resulted in significantly higher *CPT1α* gene expression (Figure 6E). In the HFD control group, *IRS2* gene expression was significantly lower than in mice on LFD and was significantly increased by treatment with PJ extract (*p* < 0.01) (Figure 6F).

## 3. Discussion

C57BL/6 mice are susceptible to diet-induce obesity and exhibit metabolic abnormalities such as dyslipidemia and hyperglycemia that phenotypically resemble obesity and type 2 diabetes [11,12]. In our study, the HFD mice treated with water also had significantly higher body weight and blood glucose compared to the LFD mice. Both the PF and PJ extracts showed anti-obesity and hypoglycemic effects in the HFD C57BL/6 mice, where administration of PF and PJ extracts (40 mg/kg body weight) reduced body weight by 9.21% and 20.27%, respectively. Liver and adipose weights, fasting glucose, TG, LDL-c, insulin, and leptin levels all decreased significantly, while serum HDL-c, adiponectin, and oral glucose tolerance levels were increased significantly in mice treated with either two fruit extract. Shim *et al.* reported that an aqueous extract of immature fruit of *P. trifoliata* (200 mg/2 mL/animal/day) suppressed body weight gain by 6% in Sprague-Dawley rats after 10 weeks [10]. However, there was no significant change in plasma TG and other serum biochemical parameters and relative organ weight. In addition, administration of the same fruit extract at low dosage of (20 mg/animal/day) did not reduce body weight gain [10]. The bioactivities of fruit extracts are due to the interaction of different bioactive compounds. Fruit at different maturities or different fruit tissues have different bioactive compounds compositions, which may have resulted in different overall bioactivities.

There was no significant difference in food intake between mice in the PF and PJ groups and the HFD group treated with water (Table 3), indicating that anti-obesity effects of PF and PJ extracts were not caused by suppressing appetite. In addition, none of the animals fed PF and PJ extracts showed abnormal clinical signs during the whole experiment, suggesting that consumption of PF and PJ was safe.

Nowadays, flavanones are attracting more and more attention in relation to NCDs. Taking naringin for example, it attenuated obesity, dyslipidemia and insulin resistance in HFD C57BL/6 mice [13]. In other research, naringin was found to attenuate insulin resistance, β-cell dysfunction, hepatic steatosis and kidney damage in a type 2 diabetes rat model [14]. Administration of naringin at approximately 100 mg/kg/day in Wistar rats resulted in 1.5% reduction in body weight after 16-week experiment compared to rat fed only with high-fat diet [15]. In addition, in a clinical study, naringin supplementation lowered plasma lipids in hypercholesterolemic subjects [16]. Narirutin from citrus peels was reported to attenuate alcoholic liver disease, and it significantly suppressed the TG and TCH in the ethanol-treated ICR mice [17]. Poncirin from *P. trifoliata* prevented adipocyte differentiation in mesenchymal stem cells [18]. In the present study, PJ extract showed a greater effect than PF extract on parameters such as body weight, organ weight, serum TG, TCH, epididymal adipocyte size, leptin and adiponectin. This difference was correlated with the higher total flavanone content in PJ extract (184.91 mg/g), compared with PF (99.21 mg/g). Therefore, although there are other bioactive compounds in the extracts such as terpenoids *etc.*, it seems reasonable to propose that flavanones play an important role in the anti-obesity and hypoglycemic effects of *P. trifoliata* extracts. In addition, naringin and poncirin were major flavonoids in two fruit extracts. Doses of naringin given to the mice from PF and PJ extracts were 3.1 mg/kg body weight and 2.0 mg/kg body weight, respectively. Doses of poncirin given to the mice from PF and PJ extracts were 0.2 mg/kg body weight and 4.3 mg/kg body weight, respectively. Our results showed that PF and PJ extracts reduced body weight by 9.21% and 20.27%, respectively. Therefore, suppression of obesity by small dose of flavanones was found in the present study. Further investigation into the action or interaction of different flavanones responsible for the anti-obesity effect is required.

Insulin, a hormone related to lipid metabolism, contributes to the development of obesity [19]. In the present study, an increase in serum insulin and HOMA-IR levels suggested there was insulin resistance in the HFD C7BL/6 mice (Figure 5A,B). Consumption of PF and PJ ameliorated insulin resistance (Figure 5A,B), suggesting that improved insulin sensitivity might partially explain the anti-obesity effects of the two fruit extracts.

Both leptin and adiponectin are closely related to obesity [20]. Leptin is mainly produced in adipocytes and its levels may reflect lipid content in the body [21]. In our study, the HFD mice treated with water exhibited hyperleptinemic symptoms, which was consistent with the diet-induced resistance to leptin action as reported previously [21,22,23]. Treatments with *P. trifoliata* extracts lowered the leptin levels (Figure 5C), which coincided with the change in the size of white adipose tissue (Figure 3B,C). Leptin secretion could be regulated by the accumulation of fat in adipocytes, which resulted in insulin resistance as observed in the obese animal model [22]. Similar observation was shown by Maeda *et al.* [23], where dietary fucoxanthin and fish oil down-regulated adipocytokines including leptin and attenuated weight gain of white adipose tissue in mice.

Adiponectin is also closely related to insulin sensitivity and it stimulates glucose uptake in muscle and inhibits hepatic glucose production [24]. Administration of adiponectin has been shown to ameliorate hyperglycemia and hyperinsulinemia in an insulin-resistant mouse model [25]. Grape seed extract increased plasma adiponectin level and lowered HOMA-IR in HFD hamsters, which resulted in lowered glycemia and inhibited obesity development [26]. Our study also showed increased adiponectin levels in mice treated with each fruit extracts (Figure 5D), which might contribute to the decreased HOMA-IR. Thus, promoting the secretion of adiponectin, reducing insulin resistance and leptin level may be an important mechanism for the anti-obesity and hypoglycemic effects of *P. trifoliata* extracts.

Type 2 diabetes, is related to either insulin resistance or impaired insulin secretion [27]. In the present study, the insulin-resistant HFD mice had impaired glucose tolerance, as shown in OGTT (Figure 4B). *P. trifoliata* extracts reduced insulin resistance as indicated by reduced insulin and HOMA-IR levels (Figure 5A,B), and the fruit-extract-treated HFD mice showed lower blood glucose levels than those of water-treated HFD mice at 60, 90 and 120 min during the OGTT (Figure 4B). Gene expression results also showed that *IRS2* gene was induced by the fruit extracts (Figure 6F), which further favored increased insulin sensitivity in the HFD mice treated with the fruit extracts.

Among the various fatty acid metabolism target genes, *FAS*, *SCD1*, and *ACCα* are involved in the biosynthesis of fatty acids while *ACOX* and *CPT1α* are related to fatty acid oxidation [28]. SCD1 is a δ-9 fatty acid desaturase required for the biosynthesis of monounsaturated fatty acids, which are key substrates for the formation of complex lipids such as TG, cholesterol esters, and phospholipids. *SCD1* has emerged as one of the key regulators in lipid and sugar metabolism, where it can affect diabetes, insulin resistance, hyperlipidemia, *etc.* [29]. Results of the present study showed that the transcript level of *SCD1* was significantly inhibited by treatments of the HFD mice with either of the fruit extracts. FAS is an important multifunctional enzyme related to lipid metabolism [30]. PF and PJ treatment resulted in a significant reduction in FAS gene expression in the liver of C57BL/6 mice. Meanwhile, transcripts of *ACCα* were reduced while those of *ACOX* and *CPT1α* were increased by both fruit extracts, which might be closely related to the lipid-lowering phenotype in the liver of C57BL/6 mice. Therefore, consumption of PF and PJ extracts might modulate lipid metabolism by affecting hepatic fatty acid synthesis and oxidation.

## 4. Materials and Methods

### 4.1. Chemicals

Acetonitrile and naringin were purchased from Sigma-Aldrich (St. Louis, MO, USA). Narirutin, didymin and poncirin were the products of J & K Scientific Ltd. (Shanghai, China). Double-distilled water (ddH_2_O) was used in all experiments. All the other reagents were of analytical grade bought from Sinopharm Chemical Reagent Co., Ltd. (Shanghai, China).

### 4.2. Fruit Materials and Preparation of P. trifoliata Extracts

Mature *P. trifoliata* fruits were collected in October 2013 from Taizhou, Zhejiang Province, China. The fruit samples were botanically authenticated by Dr. Changjie Xu from Zhejiang University (Zhejiang, China). The fruits were separated into two parts: PF and PJ. Each part of the fruit tissue was lyophilized and ground in a laboratory mill. The methods of preparing extracts were based on Zhang *et al.* with some modifications [31]. The ground powder of the two fruit parts was extracted with 20 mL of 80% methanol for 30 min with ultrasound, frequency 60 kHz and power 30 W, respectively. The samples were extracted twice and centrifuged at 10,000 rpm for 10 min. The supernatant solution was evaporated with a rotary evaporator under reduced pressure and dissolved in ddH_2_O. The aqueous solution was then purified on a C18 Sep-Pak^®^ cartridge (12 cc/2 g, Waters, Milford, MA, USA) to remove the sugar, acid and other polar substances. The cartridge was activated with 20 mL methanol, conditioned with 20 mL water, and aqueous solution was loaded onto the cartridge, which was then washed with 40–80 mL water and thoroughly dried. Compounds adsorbed to the cartridge were eluted with methanol and vacuum-dried (Concentrator Plus, Eppendorf, Germany) to obtain the PF and PJ extracts for further chemical analysis and animal experiments.

### 4.3. LC-MS and HPLC Analysis

Identification of the main flavanones in different fruit tissues was performed using an Agilent 1290-6460 Triple Quadrupole LC-MS system (Agilent Technologies Inc., Santa Clara, CA, USA). Analytical identification was performed using multiple reaction monitoring (MRM) and electrospray ionization in negative mode for the flavanones. Chromatographic separations were done on an ODS C18 analytical column (4.6 mm × 250 mm) using an Agilent 1290 Infinity HPLC system (Agilent Technologies, Santa Clara, CA, USA). The eluent was split and approximately 0.3 mL/min was introduced into the mass detector. The operation conditions were as follows: capillary 3500 v (negative), nebulizer 45 psi, dry gas flow rate 5 L/min at 325 °C. An Agilent MassHunter Workstation (Santa Clara, CA, USA) was used for data acquisition and processing.

Flavanones in different fruit tissues were analyzed by HPLC according to Sun *et al.* with some modifications [32]. Briefly, an HPLC system (2695 pump, 2996 diode array detector, Waters, Milford, MA, USA) coupled with an ODS C18 analytical column (Sunfire, 4.6 mm × 250 mm, i.d., 5 μm, Waters) was used with the detection wavelength of 280 nm. The mobile HPLC phase consisting of ddH_2_O (A) and acetonitrile (B) was performed as follows: 0–15 min, 20% B, 15–35 min, 20%–60% B, 35–40 min, 60%–100% B, 40–42 min, 100% B, 42–45 min, 100%–20% B, 45–50 min, 20% B. The separation temperature was set at 25 °C and the flow rate was 1 mL/min. Flavanones were analyzed according to the retention time and UV pattern compared with their standards.

### 4.4. Animals and Diets

All the experimental procedures were conducted following the Guide for the Care and Use of Laboratory Animals of the National Institutes of Health. This study was approved by the Committee on the Ethics of Animal Experiments of Zhejiang University (Permit Number: SYXK 2012-0178).

Forty male C57BL/6 mice were purchased from Shanghai Slac Laboratory Animal Co., Ltd. (Shanghai, China). They were at 6 weeks of age and kept in a specific pathogen free facility. Three or four mice were kept in each individual cage under 12 h light/dark cycle and fed food and water *ad* *libitum* during the entire experiment. After 7 days of adaptation, mice were then randomly split into four groups and fed specific diets for a period of 13 weeks. The groups included: (1) ten C57BL/6 mice fed with LFD (10% fat) provided by Jiangsu Medicience Biomedicine Co., Ltd. (Yangzhou, China) and water by gavage; (2) ten C57BL/6 mice fed with HFD (45% fat) provided by Jiangsu Medicience Biomedicine Co., Ltd. (Yangzhou, China) and water by gavage; (3–4) 20 C57BL/6 mice fed with HFD diet (each group contained 10 mice), and PF and PJ extracts (40 mg/kg body weight, respectively) by gavage. Before feeding to the animal, these extracts were dissolved in water at the concentration of 2 mg/mL. The dose was chosen according to a preliminary experiment. The human-equivalent dose based on body surface area is about 3.2 mg/kg body weight, respectively [33]. The extracts were given to mice once a day and six days each week. Body weight measurement started from the first week of the study and continued weekly for the entire experiment of each mouse. After 13 weeks, the mice were sacrificed by decapitation after overnight fasting. Blood samples, liver, epididymal, and perirenal adipose were collected, weighed and then stored at −80 °C.

### 4.5. Serum and Histological Analysis

Serum TG, TCH, HDL-c, and LDL-c were determined by Roche Cobas 8000 modular analyzer series (Roche Diagnostics, Basel, Switzerland). Serum insulin, leptin and adiponectin levels were analyzed by immunoassay using ELISA kits (Shanghai Lengton Biological Technology Co., Ltd., Shanghai, China) according to the manufacturer’s protocols. HOMA-IR was assessed according to a previously described method and as follows: HOMA-IR = serum glucose level (mmol/L) × serum insulin level (mU/L)/22.5 [34].

Liver and epididymal white adipose tissue samples were fixed with 4% formalin, stained with hematoxylin and eosin (H&E) and then examined under an Olympus microscope equipped with a CCD camera (DP20, Tokyo, Japan) using the DP2-BSW image analysis software system (Olympus, Tokyo, Japan). For measurement of the relative adipocyte size, adipocytes were randomly chosen and used for measurements and calculations.

### 4.6. Fasting Glucose and OGTT

Fasting blood glucose was measured every 2 weeks from week 8 of the experiment using One-Touch Ultra ZSJ 843ETT Glucometer (Johnson & Johnson, New Brunswick, NJ, USA). OGTT was conducted in week 12. All the mice fasted overnight before the test and then fed with water or *P. trifoliata* extracts by gavage. The mice were given 4 g/kg glucose orally for OGTT. Blood samples were collected from the tail vein for measurement of basal blood glucose levels (0 min) before the intake of glucose. Additional blood glucose levels were measured at 30, 60, 90 and 120 min.

### 4.7. Quantitative Real-Time PCR

Total RNA from liver was extracted with Trizol (Invitrogen Life Technologies, Carlsbad, CA, USA) according to the manufacturer’s protocol. For each treatment group, four biological replicates were used for RNA extraction. The trace contaminating genomic DNA in total RNA was removed with TURBO DNase (Ambion, Austin, TX, USA). cDNA synthesis was initiated from 1.0 μg DNA-free RNA, using iScriptTM cDNA Synthesis Kit (Bio-Rad, Hercules, CA, USA). Real-time PCR was carried out using a CFX96 instrument (Bio-Rad). The PCR protocols used SsoFast EvaGreen Supermix kit (Bio-Rad). β-Actin, a housekeeping gene, was used as the internal control. The 20 μL reaction mixture was prepared as follows: 10 μL SYBR Green Quantitative PCR Mix (Bio-Rad), 1 μL of forward primer (10 μmol/L), 1 μL of reverse primer (10 μmol/L), and 2 μL of cDNA. The real-time PCR conditions were as follows: 95 °C for 30 s followed by 45 cycles at 95 °C for 10 s, 60 °C for 30 s. The primers used in the experiments were shown in supplemental Table 4.

### 4.8. Statistical Analysis

All the data were analyzed using SPSS 17.0 statistical software (SPSS Inc., Chicago, IL, USA). In addition, the mean ± SEM for each group was calculated. Differences indicated in the figures were based on Student’s *t*-test between the LFD group or treatment groups and the HFD group treated with water, where differences were considered significant at *p* < 0.05 level.

## 5. Conclusions

In conclusion, the present study showed that extracts from two parts of *P. trifoliata* fruit had anti-obesity and hypoglycemic effects in the HFD C57BL/6 mice. Consumption of PF and PJ extracts at 40 mg/kg body weight reduced body weight by 9.21% and 20.27%, respectively. The two fruit extracts could reduce serum and liver lipid profiles and serum insulin and leptin levels, and enhance serum adiponectin level in the HFD C57BL/6 mice. In addition, fasting glucose was decreased and oral glucose tolerance levels were increased by both fruit extracts. Considering the fact that natural products are safer and contain many structurally diverse bioactive compounds capable of regulating different metabolic pathways in NCDs, the *P. trifoliata* fruit extracts may have great potential for developing functional foods or drugs to prevent obesity, type 2 diabetes, and related disorders in the future.

## Figures and Tables

**Table 1:** Structural and chromatographic characteristics of four main flavanones in *P. trifoliata* extracts.

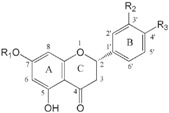	**No.**	**Flavanone**	**Rt (min)**	**UV-Peak (λ/nm)**	**R_1_**	**R_2_**	**R_3_**	**[M − H]^−^ (*m*/*z*)**	**MS/MS (*m*/*z*)**
	1	Narirutin	12.73	283.0, 330.6	rutinose	H	OH	579	151,271,313
	2	Naringin	15.3	283.0, 328.2	neohesperidose	H	OH	579	151,271,313
	3	Didymin	24.91	283.0, 329.4	rutinose	H	OCH_3_	593	285,309
	4	Poncirin	25.41	283.0, 328.2	neohesperidose	H	OCH_3_	593	285,309

**Table 2:** Contents of four main flavanones in *P. trifoliata* flavedo (PF) and juice sacs (PJ) extracts.

No.	Flavanone	PF (mg/g)	PJ (mg/g)
1	Narirutin	14.92 ± 1.94	9.43 ± 1.84
2	Naringin	77.05 ± 8.16	50.63 ± 6.76
3	Didymin	1.40 ± 0.74	17.38 ± 1.71
4	Poncirin	5.84 ± 1.28	107.46 ± 12.82
Total		99.21	184.91

**Table 3:** Effects of PF and PJ extracts on weight of different tissues and serum lipids in high-fat diet (HFD) C57BL/6 mice.

Parameter	LFD + Water	HFD + Water	HFD + PF	HFD + PJ
Weight of tissues				
Liver (g)	0.96 ± 0.03 **	1.15 ± 0.05	0.91 ± 0.02 ***	0.85 ± 0.05 ***
Epididymal adipose (g)	0.21 ± 0.02 **	0.56 ± 0.08	0.39 ± 0.02 *	0.25 ± 0.04 **
Perirenal adipose (g)	0.05 ± 0.01 *	0.18 ± 0.04	0.11 ± 0.01	0.06 ± 0.01 *
Serum lipids				
TG (mmol/L)	1.00 ± 0.09 *	1.95 ± 0.35	0.88 ± 0.05 **	0.64 ± 0.03 **
TCH (mmol/L)	2.96 ± 0.04	3.01 ± 0.12	2.88 ± 0.13	2.69 ± 0.13
HDL-c (mmol/L)	2.24 ± 0.09 **	1.42 ± 0.20	2.22 ± 0.09 **	2.21 ± 0.14 **
LDL-c (mmol/L)	0.49 ± 0.02 *	0.65 ± 0.06	0.42 ± 0.04 **	0.46 ± 0.03 *

Data are presented as mean ± SEM (*n* = 6–10). * *p* < 0.05, ** *p* < 0.01, *** *p* < 0.001, compared to the HFD group treated with water. LFD: low fat diet; TG: triglyceride; TCH: total cholesterol; HDL-c, high-density lipoprotein cholesterol; LDL-c: low-density lipoprotein cholesterol.

**Table 4:** Primers used in quantitative real-time PCR.

Gene	Forward Primer (5’ to 3’)	Reverse Primer (5’ to 3’)
*FAS*	CTGCGGAAACTTCAGGAAATG	GGTTCGGAATGCTATCCAGG
*SCD1*	TCTTCCTTATCATTGCCAACACCA	GCGTTGAGCACCAGAGTGTATCG
*ACCα*	GGCCAGTGCTATGCTGAGAT	AGGGTCAAGTGCTGCTCCA
*ACOX*	CGGAAGATACATAAAGGAGACC	AAGTAGGACACCATACCACCC
*CPT1α*	AGGACCCTGAGGCATCTATT	ATGACCTCCTGGCATTCTCC
*IRS2*	GCTCCCTGTTCCTGCAGCGG	CAAAGGTGCCAGCCCCTGGG
*β-Actin*	ATGTGGATCAGCAAGCAGGA	AAGGGTGTAAAACGCAGCTCA
